# Acute caffeine supplementation in combat sports: a systematic review

**DOI:** 10.1186/s12970-018-0267-2

**Published:** 2018-12-29

**Authors:** Luis M. López-González, Antonio J. Sánchez-Oliver, Fernando Mata, Pablo Jodra, Jose Antonio, Raúl Domínguez

**Affiliations:** 1 Nutriscience, Córdoba, Spain; 2 Area of Human Motricity and Sports Performance. Faculty of Education Sciences, Seville University, c/Pirotecnia s/n, Seville, Spain; 3 Sport Departament. School of Sport Sciences, Pablo de Olavide University, Seville, Spain; 4 Department of Education Sciences, Universidad de Alcalá, Alcalá de Henares, Spain; 5 Department of Health and Human Performance, Nova Southeastern University, Miami, Florida USA; 6 Faculty of Health Sciences, Universidad Isabel I, Burgos, Spain

**Keywords:** Ergogenic aid, Sport performance, Sport supplements, Athlete

## Abstract

Caffeine used as a supplement has been shown to improve physical and cognitive performance in several sport modalities due to its effects on the central nervous system. This review assesses the direct effects of caffeine supplementation on performance in combat sports. Using the PRISMA (Preferred Reporting Items for Systematic Review and Meta-Analysis) guidelines, relevant studies were identified through the Medline, Scopus and SPORTDiscus databases. Of 1053 search results, only 9 articles fulfilled the inclusion criteria. Of these, three studies detected no ergogenic effect of caffeine supplementation, while six studies did observe a significant positive effect. Supplementation with 3–6 mg/kg of caffeine was found to increase the glycolytic contribution to energy metabolism during the execution of real or simulated combats, as indicated by elevated blood lactate concentrations. Caffeine intake was also noted to improve levels of strength, power and upper arm muscular endurance. These effects were not paralleled by an increase in the exertion perceived by the athlete.

## Introduction

Combat sports represent around 25% of all Olympic game medals and encompass a wide variety of contact sports disciplines in which two opponents of similar physical characteristics confront each other with the objective of disabling the opponent or scoring more points than the opponent [1]. To ensure fair play, competitors must show similar levels of strength, power and agility and are therefore divided into body weight categories [2–4]. Combat sports resemble sport modalities of intermittent dynamics, in which short bursts of maximum intensity actions are interspersed with lower intensity actions [5]. For example in judo, high-intensity efforts lasting 15 to 30 s are interspersed with 5 to 10 s pauses [6]. Hence, combat modalities require a substantial contribution from both oxidative energy metabolism [7] and non oxidative metabolism (glycolysis and high-energy phosphagen system) during bouts of high-intensity actions [8].

Anthropometric requirements for combat sports are long extremities and low body fat levels [9], as well as a highly developed capacity for oxidative and non oxidative energy metabolism [10, 11]. Other important characteristics are maximal strength, power and muscle resistance [12, 13], especially hand grip strength [14, 15], arm muscle endurance [16], and reaction speed [17].

Besides these performance variables, we should not forget the central role of the glycolytic system in this type of sport [3], which gives rise to increased H^+^ production resulting in a decrease in both intramuscular and blood pH [18]. To maintain an adequate acid-base balance, various physiological adaptations occur, which can compromise performance. For instance, at the level of the central nervous system, there is an increase in subjective perceived exertion [19], while at the metabolic level, phosphofructokinase activity is inhibited, therefore impairing glycolysis [20] and phosphocreatine resynthesis [21], which leads to decreased muscle contractility [22]. As expected, these responses will compromise muscular strength and power output [23], hence reducing the capacity for repeated high intensity efforts, which is of vital importance for combat sports [24].To compete at the lightest possible weight category and thus fight against a lighter (easier) oponent, some combat athletes will opt for rapid weight loss [25]. The proportion of combat athletes who employ rapid weight loss methods (> 5% body weight) before a competition has been estimated at 86% [26]. These rapid weight loss methods include fasting during the few days before a competition combined with other techniques such as exercising with plastic wraps, self-induced vomiting and the use of laxatives and slimming pills [27]. These strategies lead to impaired thermoregulation, hormone and hydroelectric imbalances, cardiovascular stress, altered immune function and the depletion of energy stores. This last factor will compromise glycolytic yield, a performance indicator in combat sports [28], and also affect high intensity efforts [29].

Because sports at the elite level can be highly competitive, improvements as small as 0.6% are sufficient to make a difference in a competition [30]. Thus, many athletes consume dietary supplements to try to optimize their performance [31]. However, only creatine, sodium bicarbonate, β-alanine, nitrate and caffeine have shown a high level of scientific evidence for performance improvement [32]. Further, the ergogenic effects of each supplement are conditioned by the type of effort executed [33].

Caffeine (1,3,7-trimethylxantine) is present in food products such as coffee, cocoa, drinks or gels and energy bars, and can also be taken as a pharmacological supplement. Blood caffeine levels rise 15–45 min after intake [34] and peak after 60 min [35]. The caffeine molecule is similar to adenosine and competes with it, binding to adenosine receptors A_1_ and A_2a_ [36]. This means that caffeine is a potent modulator of central nervous system activity [37], inhibiting parasympathetic nervous system activity. Consequently, at the central level, caffeine supplementation increases alertness and enhances mood, reducing an individual’s rating of perceived exertion (RPE) and improving cognitive performance [38–40]. At the metabolic level, caffeine leads to elevated blood norepinephrine levels [41], which increases heart rate –both when resting [42] and during physical activity [43] – and enhances glycolytic activity [44], therefore increasing muscle energy supply during exercise [45]. At the neuromuscular level, caffeine increases the recruitment of motor units [46]. In vitro studies have shown that calcium release from the sarcoplasmic reticulum is also increased after an action potential [44], which translates to improved intra- and intermuscle coordination [47].

The central and peripheral effects of caffeine supplementation have determined its observed benefits in improving psychomotor function manifested as improved agility and decision making [48–50]. These latter factors act as performance variables in combat sports and are required during the intermittent high-intensity efforts needed for velocity and precision in rugby [51] or football [52] players. Moreover, it has been recently reported that caffeine supplementation has an ergogenic effect on specific performance in sport regardless of the dose employed [53]. In view of these findings, we here review studies that have addressed the effect of caffeine supplementation on performance variables in combat sports.

## Materials and methods

The present systematic review followed the Preferred Reporting Items for Systematic Reviews and Meta-Analysis (PRISMA) guidelines [54].

### Search strategy

The literature was explored using the databases Medline, Scopus, and SPORTDiscus, including all articles published between 2010 and 7 November 2018. Since no previous review has been conducted on this topic, the search was not limited by publication language. The search strategy used was (concept 1) (caffeine OR coffee) AND (concept 2) (supplement OR supplementation OR “ergogenic aid”) AND (concept 3) (sports OR exercise OR combat OR boxing OR judo OR karate OR effort OR “martial arts”).

### Study selection: Inclusion and exclusion criteria

Articles written in English, Spanish or Portuguese were included if they reported on randomized trials with a control group in which caffeine supplements and placebo were administered to a population of combat sport athletes in test sessions in a random manner. Also, in the test sessions, studies were required to include measurement of a performance variable in a combat simulation task and/or measurement of a performance variable specific to the modality practised.

After two investigators read the study titles and abstracts, a series of exclusion criteria were applied to reject studies that were not randomized, not double-blind with a control condition, not related to nutrition and diet, not including a caffeine supplementation protocol, along with those conducted in animals or in athletes of non-combat sport modalities.

### Data extraction and synthesis

The following information was compiled for each study: author, date of publication, sample size, participant characteristics, supplementation protocol, performance variables assessed and results. All variables in which a difference was detected related to caffeine supplementation versus control condition (placebo) are provided for the two conditions along with the % improvement recorded for the supplement calculated using the equation: (value with supplementation – value with placebo)/value with placebo × 100.

### Methodological quality

To assess the methodological quality of the studies included in this systematic review, the PEDRo scale was used. Two investigators independently assessed each study according to all 11 items of the scale. If the scores awarded by the two investigators differed, a third blinded investigator assessed the study in question.

## Results

### Study selection

**Fig. 1:**
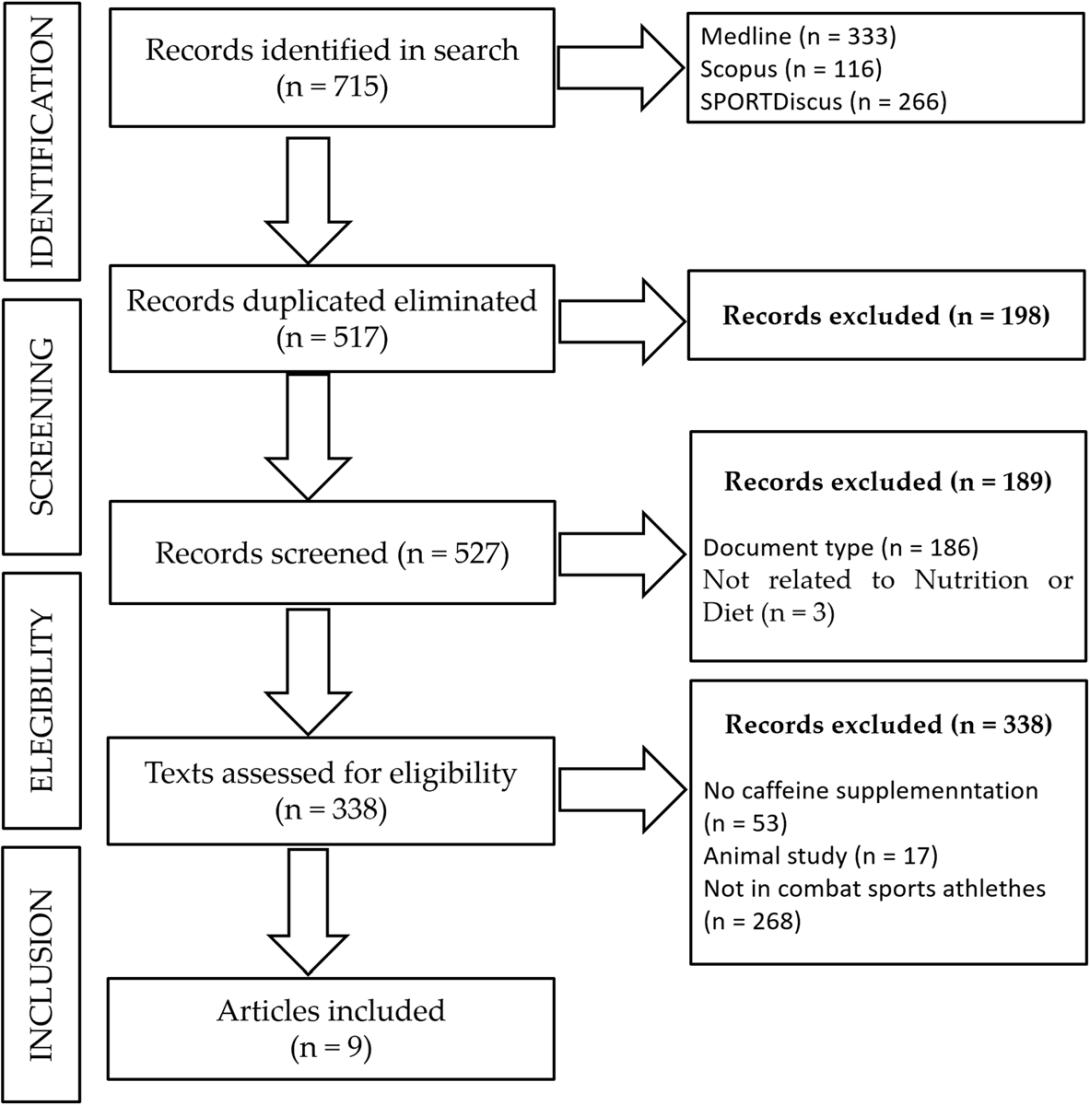
Article selection process. The present systematic review followed the Preferred Reporting Items for Systematic Reviews and Meta-Analysis (PRISMA) guidelines [54]

Out of 1053 articles identified, 715 were published after 2010. After eliminating duplicate articles (*n* = 198) and those reporting on studies with no intervention (*n* = 186) or not related to the topic (*n* = 3), 338 full-text articles were identified for this review, of which only 9 (8 published in English and 1 in Spanish) remained after applying the exclusion criteria (see Fig. 1).

### Study characteristics

The total number of study participants in the 9 studies reviewed was 109. Of these, 42 were Brazilian jiu jitsu athletes, 33 were taekwondo athletes and 34 were judo athletes. Participants were shared across three trials focusing on each of these combat sports.

The caffeine doses tested in the trials were 3 mg/kg [55, 56], 4 mg/kg [57], 5 mg/kg [58–60] and 6 mg/kg [6, 61, 62]. Supplements were ingested at different times before exercise testing: 30 min [58], 50 min [60] or 60 min [6, 55–57, 59, 61, 62]. In one study, the effects of both caffeine and caffeine plus sodium bicarbonate were examined [6].

Several tests were used to assess performance. Díaz-Lara et al. [56] analyzed the effect of caffeine supplementation using a battery of tests designed to assess variables related to taekwondo performance. Astley et al. [57] used a specific judo performance test, or special judo fitness test (SJFT), while others assessed performance in a set of 3 repetitions of a specific test such as SJFT [6, 62] or rounds of 1 taekwondo combat [59] or 2 jiu-jitsu combats [55]. Aedma et al. [63] simulated a combat-type effort on an arm ergometer. Other studies assessed fatigue through loss of performance using a set of performance variables or after a fatiguing effort resembling competition demands [55, 60, 61].

### Study results

Table 1 provides details and results of the 9 studies reviewed. In 3 of the studies, no effects of caffeine supplementation on performance were observed [58, 59, 62], in 5 an ergogenic effect was detected [55–57, 60, 61], and in the remaining study [6], a beneficial effect was reported of supplementation with caffeine plus sodium bicarbonate.

**Table 1:** Summary and results of the nine studies reviewed examining the impacts of acute caffeine supplementation on combat sports performance

Ref.	Participants	Supplementation	Test	Results
Felippe et al. [5]	Trained judo athletes (*n* = 10)	EC1: 6 mg/kg caffeine (60 min pretest) EC2: 3 × 100 mg/kg NaHCO_3_ (120, 90 and 60 min pretest) EC3: 3 × 100 mg/kg NaHCO_3_ (120, 90 and 60 min pretest) + 6 mg/kg caffeine (60 min pretest) EC4: placebo	3 x SJFT	ANOVA indicated differences between treatments: SJFT1: EC3 5.17% improved number of knockdowns vs EC4 (24.4 +/- 1.5 vs 23.2 +/- 1.5) SJFT3: EC3 8.44% improved and EC2 5.33% improved number of knockdowns vs EC4 (24.4 +/- 1.0 and 23.7 +/- 1.1 vs 22.5 +/- 1,6) Mean performance (sum of 3 SJFT): EC3 5.66% improved number of knockdowns vs EC4 (72.7 +/- 3.1 vs 68.8 +/- 4.2)
Diaz-Lara et al. [48]	Elite Brazilian jiu-jitsu athletes (*n* = 14)	EC1: 3 mg/kg caffeine (60 min pretest) EC2: placebo	Díaz-Lara et al. [49] tests pre + simulated combat + Díaz-Lara et al. [49] tests (post-combat 1) + combat 2 + Díaz-Lara et al. [49] tests (post-combat 2)	Hand grip strength (pre): Dominant hand: as in Díaz-Lara et al. [49] Non-dominant hand: as in Díaz-Lara et al. [49] CMJ (pre): as in Díaz-Lara et al. (2016) Bench press 1 RM (pre): as in Díaz-Lara et al. [49] Max number of bench press reps at maximal strength load (pre): as in Díaz-Lara et al. [49] Static lift test (pre): EC1 15.8% improved (values not specified) Static lift test (post-combat 2): EC1 17.8% improved (values not specified)
Diaz-Lara et al. [49]	Elite Brazilian jiu-jitsu athletes (n = 14)	EC1: 3 mg/kg caffeine (60 min pretest) EC2: placebo	Hand grip strength CMJ 1 RM bench press Max number of bench press reps at max load Static lift test	Hand grip strength: Dominant hand: EC1 4.4% improved vs placebo (55.9 +/- 5.1 vs 53.5 +/- 3.2 kg) Non-dominant hand: EC1 4.9% improved vs placebo (50.7 +/- 4.9 vs 48.4 +/- 5.2 kg) CMJ: EC1 3.7% improved jump height vs placebo (41.7 +/- 3.1 vs 40.6 +/- 2.6 cm) Bench press 1 RM: EC1 3.0% improved vs placebo (93.3 +/- 7.5 vs 90.5 +/- 7.7 kg) Max number of bench press reps at maximal strength load: EC1 14.7% improved (25.0 +/- 8.7 vs 21.8 +/- 8.1 reps)
Astley et al. [50]	Young national competition judo athletes (*n* = 18)	EC1: 4 mg/kg caffeine (60 min pretest) EC2: placebo	SJFT	Total knockdowns: EC1 improved 31.22% vs EC2 (29.0 +/- 2.6 vs 22.1 +/- 3.4) SJFT index: EC1 reduced by 22.29% vs EC2 (12.2 +/- 0.5 vs 15.7 +/- 0.9)
Aedma et al. [51]	Brazilian jiu-jitsu practitioners and wrestlers (n = 14)	EC1: 5 mg/kg caffeine (30 min pretest) EC2: placebo	4 x arm ergometer test (6 × 15 s + 40 s rest). Rest: 30 min	Average and peak power recorded in test: significant time but no treatment differences detected by ANOVA
Lopes-Silva et al. [52]	National and/or international competition taekwondists (*n* = 10)	EC1: 5 mg/kg caffeine (60 min pretest) EC2: placebo	3 x simulated taekwondo match rounds	No differences in any of the performance variables examined.
Cortez et al. [54]	National and/or international competition taekwondists (*n* = 13)	EC1: 5 mg/kg caffeine (60 min pretest) EC2: placebo	3 x dollyo chagi circular kick pre and post test of 3 × 60 s CMJ	Dollyo chagi (pre): Reaction speed: EC1 29% improved vs EC2 (0.65 +/- 0.17 vs 0.91 +/- 0.18 s) Dollyo chagi (post): Reaction speed: EC1 25% improved vs EC2 (1.04 +/- 0.13 vs 1.30 +/- 0.14 s)
Lopes-Silva et al. [55]	Trained judo athletes (*n* = 6)	EC1: 6 mg/kg caffeine (60 min pretest) following weight loss protocol EC2: placebo following weight loss protocol	3 x SJFT	ANOVA confirmed no effects according to time or treatment
Santos et al. [93]	Trained taekwondo athletes (n = 10)	EC1: 5 mg/kg caffeine (50 min pretest) EC2: placebo	2 x (5 x bandal tchagui kick + simulated taekwondo match). Rest: 30 min	Bandal tchagui kick pre-combat 1: EC1 11.9% improvement vs EC2 (0.37 +/- 0.007 vs 0.45 +/- 0.05 s) Combat 2: EC1 21% improvement in mean number of throws per round (12.20 +/- 6.71 vs 8.88 +/- 3.21 throws) Combat 1 vs combat 2: EC2 reduced throw number and time (values not specified)

*EC* experimental condition, *NaHCO*_*3*_ sodium bicarbonate, *SJFT* special judo fitness test, *CMJ* countermovement jump test

### Study methodological quality

All studies were awarded a PEDro score of 10 indicating their excellent methodological quality. In addition, none of the studies had any conflicts of interest or funding to declare such that they were not motivated by comercial interests.

## Discussion

The special judo fitness test (SJFT) is a laboratory test that assesses different technical actions of judo and has been described as valid and reliable to assess judo performance [64, 65]. Given the relationship between the number of knockdowns executed in the SJFT and performance, Lopes-Silva et al. [62] designed their study to examine the effects of caffeine supplementation (6 mg/kg) on the number of knockdowns produced in a session consisting of 3 sets of SJFT in 6 trained judo athletes. In this study, ANOVA indicated an effect of time in that RPE and blood lactate concentrations increased during the execution of the three sets. However, no effects of caffeine were produced on knockdowns, RPE or blood lactate [62]. These findings contrast with those reported by Astley et al. [57], who noted that supplementation with a caffeine dose of 4 mg/kg one hour before the test led to more knockdowns in the SJFT (31%) and to an increased SJFT index (a variable relating the number of knockdown actions to heart rate) in 18 national competition level judo athletes. The different findings of the two studies may perhaps be explained by the small sample size in the study by Lopes-Silva et al. [62], along with its heterogeneous nature, as it was composed of athletes of up to 4 different weight categories.

Lopes-Silva et al. [66] assessed the metabolic response to three taekwondo throws in a study conducted in 10 international competition level taekwondists. These authors observed that caffeine supplementation affected variables related to throw distance or number. Interestingly, they noted increased glycolytic activity following caffeine intake reflected by a significant increase in blood lactate concentrations. A possible explanation could be a reduced effect of adenosine on phosphofructokinase inhibition [44], a response that takes place in situations of acidosis as a mechanism to avoid increased glycolytic activity [20] and thus maintain the acid-base balance. Besides the beneficial enzyme effect of delaying fatigue, increased blood lactate concentrations could also reflect the greater recruitment of type II motor units during the exercise effort [67]. These motor units show a greater dependence on glycolytic metabolism [68].

In contrast, Aedma et al. [58] detected no increases in blood lactate concentrations or improved performance during four sets of a test consisting of 6 × 15 s sets on an arm ergometer performed at maximum intensity, separated by 40 s rest intervals. Only in an ANOVA was a significant effect noted of the factor time as average power output diminished across successive work sets. While this study simulated the intermittent dynamics of combat sports based on high-intensity efforts interspersed with rest periods [5], the type of test employed was very unspecific for the athletes. Given that a higher performance level increases the ergogenic potential of caffeine supplementation [69], it could be that owing to the cyclic efforts of ergometry (not resembling the acyclic efforts needed in combat sports), this ergogenic effect may have been minimized.

Another of the 9 studies reviewed here sought to assess the effect of caffeine supplementation on variables considered performance factors in Brazilian jiu-jitsu [56]. In this study, the impacts were assessed of a 3 mg/kg dose of caffeine 60 min before performing a battery of tests. These tests included hand grip dynamometry, countermovement jump test (CMJ), bench press 1 RM and the maximum number of repetitions conducted at a load at which the athletes achieve their peak power outputs. Results indicated that acute caffeine supplementation gave rise to improved maximal strength in that performance was increased at the strength levels of the hand grip and bench press 1 RM tests. This improved 1 RM for the bench press is in agreement with results reported for strength trained men [70] and women [71], while improved hand grip strength has also been observed for a higher caffeine dose (6 mg/kg) [46]. These findings are very significant, as hand strength has been linked to performance in combat sport modalities [14, 72]. Muscular endurance in the arms is also considered a performance indicator in combat sports [16]. In the study by Diaz-Lara et al. [56], it was observed that, following caffeine supplementation, participants showed a ~ 15% increase in the number of repetitions completed lifting a load at which maximum power levels are produced, consistent with findings reported for the same exercise and load (60% 1 RM) [73]. As maximal strength, power and muscular endurance are performance variables in combat sports [12, 13], the beneficial effects observed by these authors on hand grip strength, 1 RM and maximum number of bench press repetitions reflect a potential ergogenic effect of caffeine supplementation.

To obtain maximum strength levels, athletes often target the force-time relationship (force manifestation index or rate of force development [RFD]) [74]. One of the variables used to check the relationship between force and its application time is power output determined in a force/velocity analysis during strength training [75]. In the study by Diaz-Lara et al. [56], caffeine supplementation improved the average power generated during the first 15 repetitions of their weight lifting test conducted at the peak power load. These results indicate the athletes were able to lift similar loads at greater velocities. This was one of the athletes’ objectives and was also reported for a dose of 3 mg/kg of caffeine for loads exceeding 30% of 1 RM [47] and for doses of 6 and 9 mg/kg for loads of 25–90% of 1 RM [76].

In another study, Diaz-Lara et al. [56] examined responses to the previous battery of tests added to a static lift test before and at the end of two taekwondo combats separated by a 20 min rest period. According to their results, supplementation with 3 mg/kg of caffeine induced similar improvements as in their earlier study [56], and there was also a 15.8% improvement in the static lift test before the first combat. However, it was observed that the beneficial effect of supplementation on the test results only persisted in the static lift test at the end of the first match (17.8%). Further, supplementation was found to have an ergogenic effect on specific jiu jitsu performance, as the time and number of throws increased during the two combats. The increased intensity of the combats, indicated by the greater number of assault actions and time dedicated to these actions, was accompanied by higher blood lactate concentrations at the end of the combats in the experimental caffeine condition. This observation could be explained by caffeine’s glycolysis potentiating effect [44]. Such effects on specific performance support the findings reported by Astley et al. [57].

Santos et al. [60] assessed the effect of supplementation with 5 mg/kg of caffeine before undertaking 2 combats, each consisting of 3 kick throws, separated by 30 min rest periods. These authors also measured reaction speed as the time taken to complete 5 bandal tchagui kicks before and at the end of each combat. Bandal tchagui kicks are the most employed technique in taekwondo [77, 78] and consist of a single mid-section kick targeting the opponent’s abdomen [79]. The findings of this study were that caffeine supplementation had an ergogenic effect on specific performance. This determined that caffeine showed a more intense effect in the first combat, increasing the time and number of kick throws. In addition, ANOVA revealed that when the athletes took the caffeine supplement, no reduction in work intensity was produced during the combats. Given the dynamics of competitions, including multiple combats in a single day, the ability to maintain power output levels between combats is considered a performance indicator [58]. The lack of performance decline in the second combat after caffeine supplementation observed in the study by Santos et al. [60] suggests a progressive effect of caffeine intake on performance.

Another important finding of the study conducted by Santos’ group [60] was that caffeine supplementation had a positive effect in reducing the execution time of the bandal tchagui kick, which translates into an improved reaction speed as a combat sport performance indicator [17]. This beneficial effect also confirms prior reports of a shortened reaction time in response to a visual stimulus following caffeine supplementation [48, 80]. This effect of caffeine intake on reaction speed could be mediated by increased levels of circulating catecholamines [81] and by increased activity of the sodium-potassium pump, improving the sarcoplasmic availability of calcium [82]. In parallel, the antagonistic effect of adenosine could potentiate neurotransmitter release, improving motor neuron transmission [82, 83].

Similarly to Santos et al. [60], Cortez et al. [61] measured reaction speed through electromyographic monitoring during the execution of the dollyo chagi kick in taekwondists, following the intake of 5 mg/kg of caffeine before and after a fatiguing exercise (3 sets × 1 min consecutive CMJs). These authors observed that acute caffeine supplementation reduced the reaction time of the rectus femoris muscle, which plays a major role in the technical action examined [84], passing from 29% before to 25% after completing the strenuous task. Besides reflecting an improved reaction speed both in pre-exercise and fatiguing conditions, measuring an explosive effort such as the dollyo chagi kick before and after an exercise test resembles the strategy used in other studies that have assessed neuromuscular performance in a CMJ test. Effectively, this technical action of the dollyo chagi kick reflects the neuromuscular performance of the whole motor system [85]. We consider that a performance loss in an explosive effort from the start to the end of an exercise test is a symptom of neuromuscular fatigue [74, 86, 87]. If the electromyographic delay is taken as an indicator of neuromuscular fatigue, we could conclude that the investigation by Cortez et al. [61] shows that caffeine supplementation gives rise to lower levels of fatigue compared to the intake of placebo (5.5% vs 25%).

As for caffeine, a high level of scientific evidence for ergogenic effects in sport has been reported for supplements such as creatine, nitrate, β-alanine and sodium bicarbonate [32]. For this reason, athletes usually combine a few of these in their ergogenic-nutritional strategy despite scarce knowledge of their interaction effects [88]. When taken as a supplement, sodium bicarbonate increases blood pH and maintains intramuscular pH due to an increased diffusion velocity of H^+^ ions arising from glycolysis as the consequence of a higher concentration gradient [89]. Accordingly, given the proposed effects of caffeine including an increased rate of glycolysis [59] via increased catecholamine concentrations [81], the amplified recruitment of motor type II units, and impaired phosphofructokinase inhibition [44], caffeine and sodium bicarbonate taken together could have an enhanced ergogenic effect over that produced after the intake of one of these supplements alone.

To address the combined effect of sodium bicarbonate/caffeine, Felippe et al. [6] administered caffeine (6 mg/kg), sodium bicarbonate (300 mg/kg), caffeine (6 mg/kg) + sodium bicarbonate (300 mg/kg) or placebo to a group of 10 judoists before a session consisting of 3 sets of SJFT. The results of this study indicated that caffeine plus sodium bicarbonate ingestion conferred improved performance. This improvement was detected as a significant increase in the number of knockdowns in the first (5.17%) and third (8.44%) SJFT sets, and in the total number of knockdowns recorded for the whole session (5.66%) compared with the intake of placebo. These authors reported that supplementation improved performance without increasing RPE [6]. Many authors have described ergogenic effects of caffeine intake with no change [6, 59, 60] or even a concomitant decline [57] in the RPE. Such effects on the perception of exertion could be attributable to the antagonistic effects of caffeine on adenosine [36], which improves an individual’s state of alertness [90]. Further, caffeine supplements can affect emotional states associated with an adequate performance profile such as improving mood [73] and reducing the feeling of fatigue [91, 92].

Caffeine supplements, however, have been linked to a series of secondary effects. Doses such as those used in the studies reviewed here (> 3 mg/kg) have been related to an increased heart rate both in the resting state [93] and in response to high-intensity physical exercise [73, 94]. Caffeine doses as high as 6 mg/kg induce other symptoms such as raised systolic blood pressure, lack of attention, and anxiety [71]. Thus high supplementation doses may lead to diminished performance is some athletes who are particularly sensitive to these side-effects [95]. Because of the plateau effect of doses of 3–9 mg/kg [96], we would recommend the intake of 3 mg/kg in athletes starting caffeine supplementation. Further, since prolonged caffeine intake can provoke sleep alterations [97], which may in turn modify sports performance [98] and also lead to tolerance reducing its ergogenic potential [99], we recommend its use only in competitions and in athletes who have shown a good response to caffeine during training.

## Conclusions and practical applications

Caffeine doses of 3–6 mg/kg have been associated with increased glycolytic activity during real or simulated combats. This effect is accompanied by increased blood lactate concentrations and improved performance, as measured through the engagement time or number of throws performed in a contest. In addition, the higher rate of glycolysis takes place in the absence of a concomitant increase in the level of exertion perceived by the athlete. By way of conclusion, caffeine supplementation could have an ergogenic effect in combat sport practitioners and thus improve performance indicators such as hand grip strength and the strength, power and muscular endurance of the arms.

## Abbreviations

ANOVA: Analysis of variance
CMJ: Countermovement jump test
PRISMA: Preferred Reporting Items for Systematic Review and Meta-Analysis
RFD: Rate of force development
RM: Repetition maximum
RPE: Rating of perceived exertion
SJFT: Special judo fitness test
